# SERIPH: a two-step extraction protocol for selective enrichment of semi-extractable RNAs

**DOI:** 10.1261/rna.081061.126

**Published:** 2026-09

**Authors:** Naoko Fujiwara, Junichi Iwakiri, Chao Zeng, Daiki Kohsho, Kiyoshi Asai, Michiaki Hamada, Tetsuro Hirose

**Affiliations:** 1 Graduate School of Frontier Biosciences, The University of Osaka, Suita 565-0871, Japan; 2 Graduate School of Frontier Sciences, The University of Tokyo, Kashiwa 277-8562, Japan; 3 Faculty of Science and Engineering, Waseda University, Tokyo 169-8555, Japan; 4 Institute for Open and Transdisciplinary Research Initiatives, The University of Osaka, Suita 565-0871, Japan

**Keywords:** semi-extractable RNAs, AGPC, architectural RNAs, membraneless organelles, readthrough transcripts, stress responses

## Abstract

Conventional RNA extraction with acid guanidinium thiocyanate–phenol–chloroform (AGPC) reagents incompletely recovers a subset of transcripts, termed semi-extractable RNAs (seRNAs). This underrepresented RNA population includes architectural RNAs (arcRNAs), which scaffold membraneless organelles, as well as stress-induced downstream-of-gene readthrough transcripts (DoGs). Although our previously developed AGPC extraction method incorporating physical disruption, such as heating or needle shearing, enhances seRNA recovery, it also co-extracts abundant, readily extractable RNAs, resulting in mixed populations that hinder precise characterization. Here, we present SERIPH (semi-extractable RNA isolation from the interphase), a simple two-step protocol that selectively enriches seRNAs by separating them from readily extractable RNA species. In SERIPH, the aqueous phase containing extractable RNAs is first removed following conventional AGPC extraction. The remaining interphase is subsequently subjected to the extraction procedure incorporating physical disruption, yielding a fraction selectively enriched for semi-extractable transcripts. Transcriptome-wide RNA sequencing demonstrates that SERIPH robustly enriches established seRNAs, including arcRNAs and stress-induced DoGs, while efficiently depleting abundant mRNAs. Compared with the previous extraction method alone, SERIPH increases both the number and genomic extent of detectable DoG loci and enhances sensitivity for weakly semi-extractable transcripts that fall below conventional threshold-based classifications. SERIPH further reveals enrichment of intron-retaining transcript subsets within the semi-extractable RNA fraction. By expanding the detectable seRNA repertoire and enabling selective enrichment, SERIPH establishes a practical framework for focused, high-resolution analysis of seRNA populations. This methodological advance facilitates comprehensive characterization of seRNAs and supports studies of RNA processing, transcriptional regulation, and RNA-mediated nuclear organization in stress responses and disease.

## INTRODUCTION

RNA extraction using acid guanidinium thiocyanate–phenol–chloroform (AGPC) reagents, such as TRIzol, has long been a cornerstone of RNA analysis in molecular biology research (Chomczynski and Sacchi 1987; Chomczynski 1993). However, standard AGPC extraction does not recover all cellular RNAs with equal efficiency. A subset of RNAs is poorly recovered under conventional AGPC conditions and requires stronger physical disruption, such as heating or needle shearing, to be efficiently extracted. This property, termed *semi-extractability*, has unveiled a previously overlooked layer of transcriptome complexity (Chujo et al. 2017).

Systematic comparisons between conventional AGPC extraction and physical disruption-assisted AGPC extraction have identified hundreds to thousands of semi-extractable RNAs (seRNAs) across multiple cell types (Chujo et al. 2017; Iwakiri et al. 2023; Zeng et al. 2023). Importantly, these seRNAs include architectural RNAs (arcRNAs), a subclass of long noncoding RNAs (lncRNAs) that serve as molecular scaffolds for membraneless organelles (MLOs) by recruiting and concentrating specific proteins, RNAs, and other macromolecules (Fujiwara et al. 2025; Hirose et al. 2025). A well-known example is NEAT1_2, the essential scaffold of paraspeckles (Hutchinson et al. 2007; Sasaki et al. 2009; Chujo et al. 2017; Yamazaki et al. 2018, 2021). By promoting biomolecular condensate assembly, arcRNAs regulate key cellular processes, and their dysregulation is linked to cancer and other diseases (Banani et al. 2017; Alberti and Hyman 2021; Hirose et al. 2023). The semi-extractability of arcRNAs likely reflects extensive multivalent interactions with protein and nucleic acid partners, suggesting that this property can serve as a useful hallmark for identifying novel condensate-forming RNAs (Fujiwara et al. 2025; Huang et al. 2025; Klobučar et al. 2025).

In addition to canonical, independently transcribed arcRNAs, *downstream-of-gene transcripts (DoGs)* represent another class of semi-extractable transcripts (Iwakiri et al. 2023). DoGs are readthrough RNAs that extend beyond normal transcription termination sites and are induced under stress conditions such as osmotic stress, oxidative stress, hypoxia, thermal stress, and viral infection (Rutkowski et al. 2015; Vilborg et al. 2015, 2017; Bauer et al. 2018; Heinz et al. 2018; Wiesel et al. 2018). Beyond stress responses, DoGs are often highly expressed in pathological contexts, including cancers, and can also be detected in normal tissues (Grosso et al. 2015; Caldas et al. 2024; Pabis et al. 2024). Although their physiological functions remain largely unclear, DoGs are thought to be closely associated with altered transcriptional and RNA-processing states (Vilborg et al. 2017; Alpert et al. 2020; Castillo-Guzman et al. 2020; Reimer et al. 2021; Rosa-Mercado et al. 2021; Morgan et al. 2022; Rosa-Mercado and Steitz 2022; Caldas et al. 2024; Pabis et al. 2024; Podszywałow-Bartnicka et al. 2025), either as a consequence or as a contributing factor, during cellular adaptation and survival in stress and recovery phases (Vilborg and Steitz 2017; Heinz et al. 2018; Hadar et al. 2022; Morgan et al. 2022; Caldas et al. 2024). Consistent with their semi-extractable properties, DoGs constitute 1,6-hexanediol-sensitive condensates, suggesting a potential link between readthrough transcription and condensate formation under stress conditions (Iwakiri et al. 2023). Together with their stress inducibility, this property makes DoGs useful for investigating how stress-responsive transcriptional dynamics are coupled to RNA processing and nuclear organization (Nemeroff et al. 1998; Dye and Proudfoot 1999; Kyburz et al. 2006; Davidson and West 2013; Davidson et al. 2014; Vilborg and Steitz 2017; Alpert et al. 2020; Castillo-Guzman et al. 2020; Reimer et al. 2021; Rosa-Mercado et al. 2021; Morgan et al. 2022; Rosa-Mercado and Steitz 2022; Caldas et al. 2024; Pabis et al. 2024; Podszywałow-Bartnicka et al. 2025). Thus, arcRNAs and DoGs exemplify biologically significant RNA classes whose semi-extractability highlights the need to develop experimental strategies to study them more effectively.

Although physical disruption-assisted AGPC extraction enabled the systematic detection of seRNAs through comparison with conventional AGPC extraction, thereby facilitating the identification of arcRNAs and DoGs, it has an important limitation: it extracts both semi-extractable and readily extractable RNAs together. The resulting mixed RNA pool complicates precise characterization of seRNAs, particularly when transcripts with different extractability properties originate from the same genomic loci.

To overcome this limitation, here we developed SERIPH (semi-extractable RNA isolation from the interphase), a two-step RNA extraction protocol designed to selectively enrich seRNAs. In SERIPH, the aqueous phase containing readily extractable RNAs generated by conventional extraction is first removed, and the remaining interphase is then subjected to the heating-based version of physical disruption-assisted AGPC extraction, hereafter referred to as heat-assisted improved AGPC extraction (hiAGPC). This strategy facilitates the focused analysis of seRNAs by minimizing contamination from abundant, readily extractable species.

In this study, we show that SERIPH robustly enriches known seRNAs, including NEAT1_2 and stress-induced DoGs, enhances detection of weakly semi-extractable transcripts overlooked by previous methods, and reveals intron-retaining transcript subsets within the semi-extractable RNA fraction. By facilitating selective and sensitive analysis of this underexplored RNA population—including potential arcRNAs—SERIPH provides a powerful framework for advancing our understanding of RNA-mediated intracellular organization, stress responses, and disease-associated RNA dysregulation.

## RESULTS

### Development of an interphase-based method to isolate seRNAs

**FIGURE 1.**
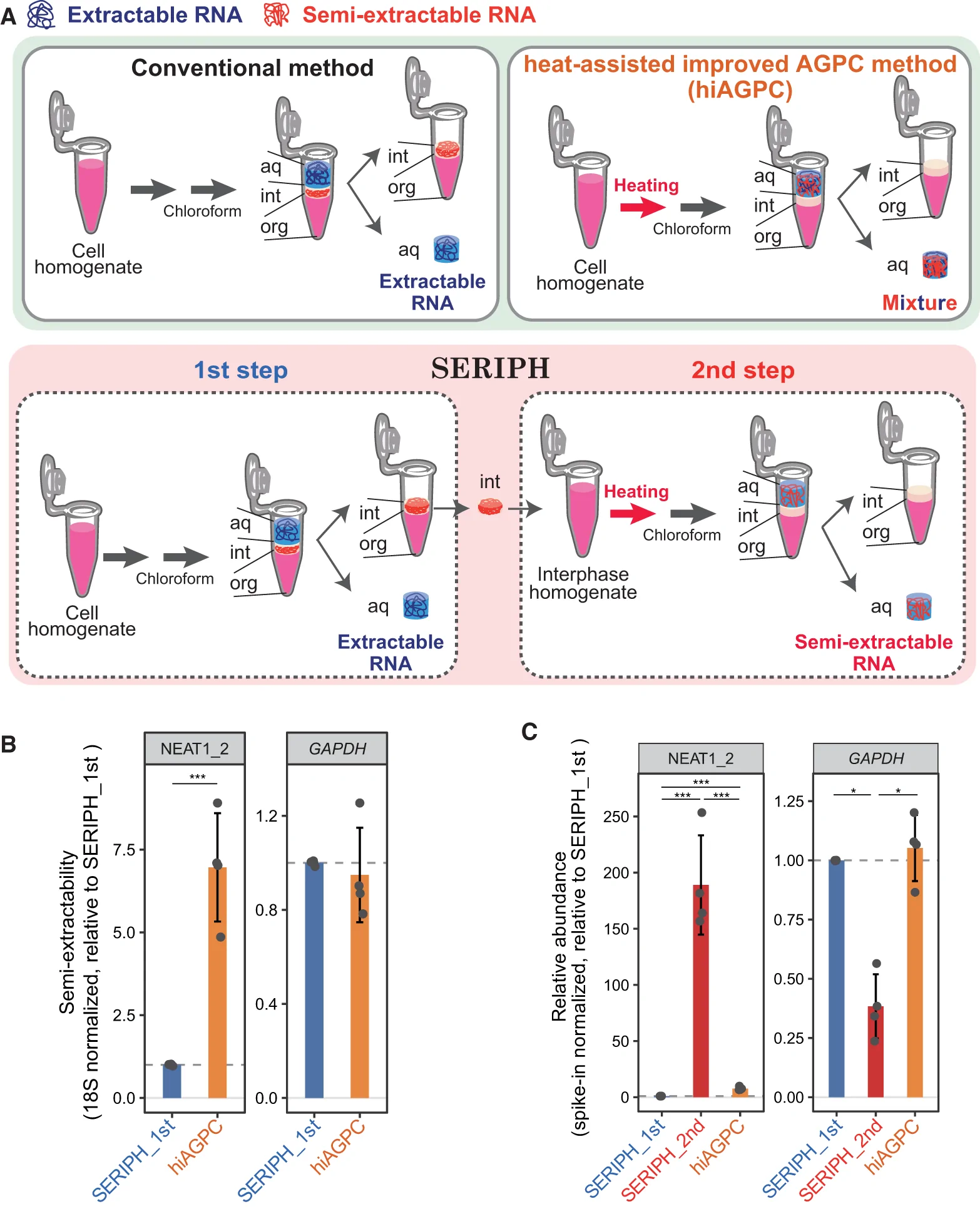
SERIPH selectively enriches semi-extractable RNAs by interphase-based fractionation. (*A*) Schematic overview of SERIPH in comparison with conventional and hiAGPC RNA extraction methods. In the conventional method, readily extractable RNAs are recovered in the aqueous phase, whereas semi-extractable RNAs remain largely retained at the interphase. hiAGPC incorporates heating at 55°C, improving recovery of semi-extractable RNAs but yielding a mixed RNA pool. In SERIPH, the aqueous phase is first removed (*SERIPH 1st step*), and the remaining interphase is then washed and subjected to the hiAGPC procedure to obtain a fraction selectively enriched for semi-extractable RNAs (*SERIPH 2nd step*). Extractable and semi-extractable RNAs are shown in blue and red, respectively. “aq,” “int,” and “org” denote the aqueous, interphase, and organic phases, respectively. (*B*) Semi-extractability analysis of NEAT1_2 lncRNA and *GAPDH* mRNA by RT–qPCR. Four technical replicates derived from the same HEK293 homogenate were analyzed. RNA abundance was measured in the SERIPH 1st-step fraction (corresponding to the conventional preparation) and the hiAGPC preparation, normalized to 18S rRNA, and expressed relative to the SERIPH 1st-step fraction. Bars represent mean ± SD, and dots indicate individual replicate values. Statistical significance was assessed by paired two-tailed *t*-tests on log_2_-transformed fold-change values with Benjamini–Hochberg correction for multiple comparisons across targets shown in Figure 1B and Supplemental Figure S2C. (*), (**), and (***) indicate adjusted *P* < 0.05, <0.01, and <0.001, respectively. (*C*) Partitioning of NEAT1_2 lncRNA and *GAPDH* mRNA across extraction methods assessed by RT–qPCR. RNA from the same HEK293 homogenates used in *B* was subjected to SERIPH 1st-step, SERIPH 2nd-step, or hiAGPC extraction. RNA abundance was normalized to an in vitro–transcribed *Cypridina* luciferase spike-in control and expressed relative to the SERIPH 1st-step fraction. Bars represent mean ± SD, and dots indicate individual replicate values. Statistical significance was assessed by repeated-measures ANOVA on log_2_-transformed fold-change values, followed by paired two-tailed *t*-tests with Benjamini–Hochberg correction. The dashed line indicates the baseline (SERIPH 1st-step = 1). (*), (**), and (***) indicate adjusted *P* < 0.05, <0.01, and <0.001, respectively.

Motivated by our previous report that NEAT1_2 can be recovered from the interphase (Chujo et al. 2017), and by phase-partitioning RNP-capture methods that accumulate RNA–protein complexes at the interphase layer (Komatsu et al. 2018; Queiroz et al. 2019; Urdaneta et al. 2019; Villanueva et al. 2020; Sztachera et al. 2025), we hypothesized that semi-extractable RNP-dense transcripts are preferentially retained in this fraction. We therefore devised SERIPH (Fig. 1A; Supplemental Fig. S1): following AGPC lysis, the aqueous phase (“SERIPH 1st-step”; readily extractable RNAs) is removed, and the remaining interphase is subjected to hiAGPC to obtain the “SERIPH 2nd-step” fraction.

Total RNA was prepared from the same HEK293 cell homogenate using either SERIPH or hiAGPC for comparison. Using our previously established semi-extractability assessment framework based on comparisons between hiAGPC and conventional extraction, RT–qPCR confirmed the expected semi-extractability of NEAT1_2, whereas *GAPDH* did not exhibit semi-extractable behavior (Fig. 1B; the SERIPH 1st-step fraction corresponds to the conventional preparation). We next examined the partitioning of these marker RNAs across the SERIPH fractions using an in vitro-transcribed spike-in control for normalization, because endogenous reference RNAs may themselves be subject to partitioning (Fig. 1C). Relative proportions were quantified to account for differences in total RNA recovery between fractions (Supplemental Fig. S2A). Compared with the conventional preparation (SERIPH 1st-step fraction), NEAT1_2 was strongly enriched in the SERIPH 2nd-step fraction, whereas enrichment was substantially weaker in the hiAGPC preparation (Fig. 1C). In contrast, *GAPDH* mRNA and 18S rRNA were markedly depleted from the SERIPH 2nd-step fraction (Fig. 1C; Supplemental Fig. S2B). Similar trends were observed for another semi-extractable marker, PVT1 lncRNA, a putative arcRNA localized in specific nuclear condensates (Supplemental Fig. S2B,C; Chujo et al., 2017). These results were reproducible in an independent cell line (Supplemental Fig. S2D,E). We also observed modest enrichment of MALAT1 lncRNA, previously reported to exhibit no obvious semi-extractable properties, in the SERIPH 2nd-step fraction, although the degree was much lower than that of NEAT1_2 and PVT1 (see Discussion; Supplemental Fig. S2B,D; Chujo et al. 2017). These results demonstrate that SERIPH separates RNA markers according to their extractability.

### SERIPH enables selective and sensitive transcriptome-wide detection of seRNAs

**FIGURE 2.**
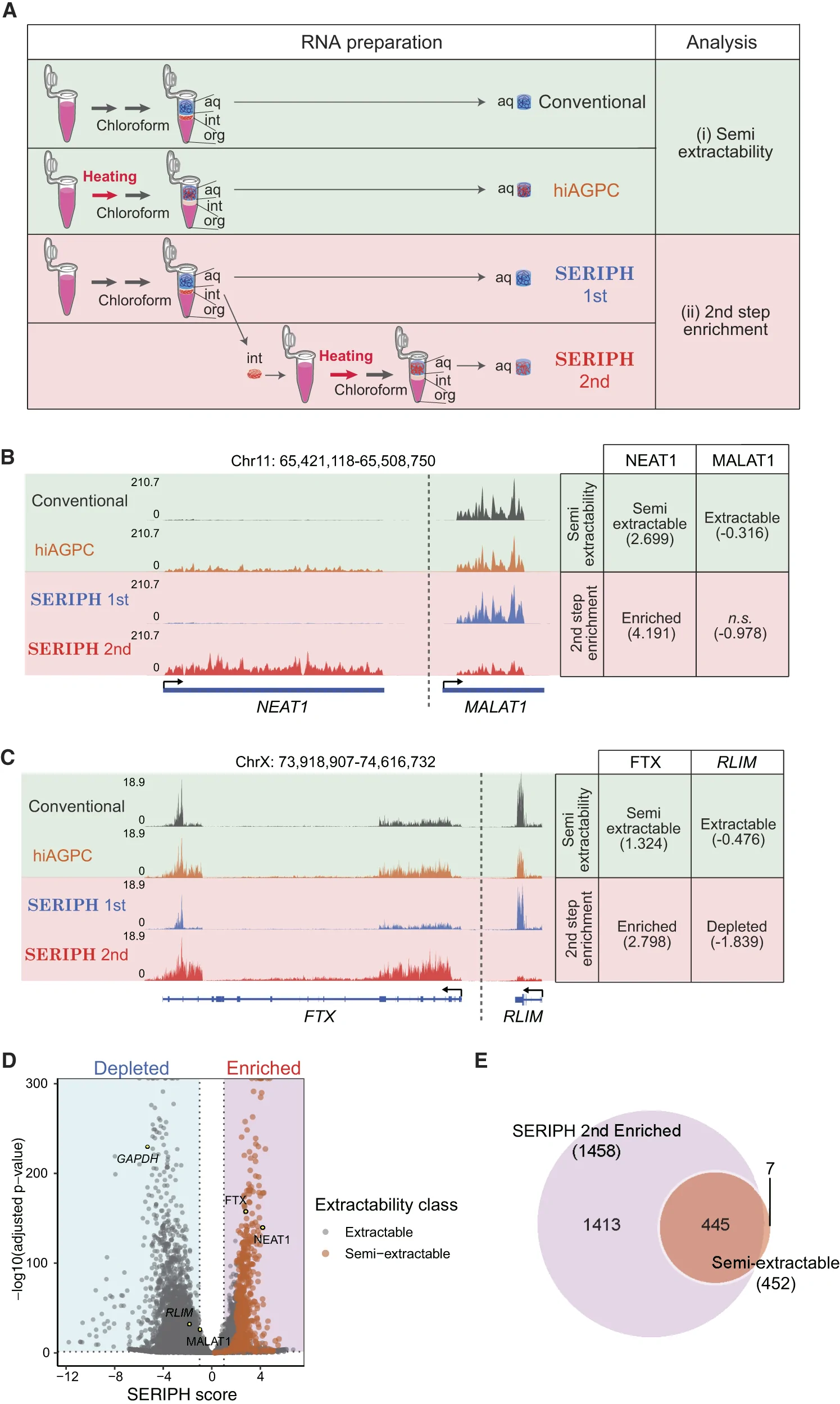
Transcriptome-wide enrichment and enhanced sensitivity for semi-extractable RNAs by SERIPH. (*A*) Overview of the transcriptome-wide analysis workflow. Short-read RNA sequencing was performed on RNAs prepared using conventional AGPC extraction, hiAGPC, and SERIPH (1st-step and 2nd-step fractions). Semi-extractability was defined by increased abundance in hiAGPC relative to conventional preparations, and SERIPH 2nd enrichment was assessed by comparing 2nd-step and 1st-step fractions. (*B*) Genome browser view of the *NEAT1*–*MALAT1* locus. Normalized coverage tracks are shown with replicates overlaid. Numbers in parentheses indicate scores from the indicated analysis. Black dotted lines denote omitted genomic regions. Black arrows indicate the transcription direction. (*C*) Volcano plot showing transcripts enriched or depleted in the SERIPH 2nd-step fraction relative to the 1st-step fraction. The *x*-axis represents the SERIPH score (2nd-step vs. 1st-step), and the *y*-axis represents −log_10_ adjusted *P*-value. Semi-extractability annotations derived from the original analysis are overlaid. Semi-extractable transcripts are shown as orange dots and extractable transcripts as gray dots. Cyan and magenta shaded regions indicate thresholds for 2nd-step-depleted and 2nd-step-enriched transcripts, respectively. Selected transcripts analyzed in Figure 2 and Supplemental Figure 2 are highlighted to show their positions within the global distribution. (*D*) Venn diagram illustrating overlap between transcripts annotated as semi-extractable in the original data set and transcripts enriched in the SERIPH 2nd-step fraction. Numbers indicate total, shared, and unique transcripts. (*E*) Distribution of semi-extractability scores for transcripts “Enriched” or “Depleted” in the SERIPH 2nd-step fraction but not classified as “semi-extractable” in the original threshold-based analysis. Boxplots show semi-extractability scores for transcripts originally classified as extractable, grouped according to their enrichment status in the SERIPH 2nd-step fraction; the distribution for originally semi-extractable transcripts is shown for comparison. The red dashed line indicates the original semi-extractability threshold (log_2_FC ≥ 1). Statistical significance was assessed using the Kruskal–Wallis test followed by Dunn's post hoc test with Holm correction for multiple comparisons. (*) *P* < 0.05, (**) *P* < 0.01, (***) *P* < 0.001.

We next evaluated the separation of seRNAs and readily extractable RNAs at the transcriptome-wide level. Short-read RNA sequencing was performed on RNAs prepared from HEK293 cells using conventional AGPC extraction, hiAGPC, and SERIPH (Fig. 2A). We assessed both (i) semi-extractability, based on RNA abundance in hiAGPC relative to the conventional AGPC preparation, and (ii) SERIPH enrichment, based on RNA abundance in the SERIPH 2nd-step relative to the 1st-step fraction (Fig. 2A; Supplemental Table S1). The log_2_ fold change between hiAGPC and conventional preparations was defined as the semi-extractability score, whereas the log_2_ fold change between SERIPH 2nd- and 1st-step fractions was defined as the SERIPH score. Transcripts with a semi-extractability score ≥1.0 and adjusted *P* < 0.05 were classified as “semi-extractable,” whereas those with a SERIPH score ≥1.0 or ≤−1.0 and adjusted *P* < 0.05 were classified as 2nd-step-“Enriched” or 2nd-step-“Depleted,” respectively.

We first examined prototypical semi-extractable lncRNA NEAT1_2 and its neighboring extractable lncRNA MALAT1 (Fig. 2B; Chujo et al. 2017). NEAT1_2 exhibited a higher semi-extractability score (2.699), consistent with a semi-extractable profile, whereas MALAT1 did not (semi-extractability score: −0.316). In SERIPH, NEAT1_2 was strongly enriched in the 2nd-step fraction (SERIPH score: 4.191), while MALAT1 was preferentially detected in the 1st-step fraction (SERIPH score: −0.987). Readily extractable mRNAs, including *GAPDH* and histone mRNAs, were strongly depleted from the SERIPH 2nd-step fraction (Supplemental Fig. S3A,B). In contrast, the lncRNA FTX, previously identified as seRNA (Chujo et al. 2017), was preferentially enriched in this fraction (semi-extractability score: 1.324; SERIPH score: 2.798; Supplemental Fig. S3C).

At the transcriptome-wide level, the SERIPH 2nd-step fraction contained distinct subsets of transcripts enriched or depleted relative to the 1st-step fraction (Fig. 2C). We overlaid semi-extractability annotations derived from the original RNA preparation data set onto this SERIPH data set. Focusing on exonic reads, 452 of 18,168 expressed genes were classified as semi-extractable, and most of these were enriched in the SERIPH 2nd-step fraction (445 of 452; Fig. 2C,D).

Notably, SERIPH also identified many 2nd-step-enriched transcripts that did not meet the original semi-extractability threshold but nevertheless showed subtle, detectable semi-extractability (Fig. 2D,E). Analysis focused on unspliced transcripts further expanded the set of 2nd-step-enriched genes and recapitulated the enrichment of semi-extractable transcripts observed in the exon-focused analysis (Supplemental Fig. S3D–F), suggesting that taking splicing information into account improves the detection of transcripts preferentially enriched in the SERIPH 2nd-step fraction (see Fig. 5).

### Selective enrichment of seRNAs by SERIPH facilitates detection of DoG readthrough transcripts

Given that SERIPH improves seRNA detection at the gene level, we next asked whether SERIPH enhances the detection of DoGs, which frequently exhibit semi-extractability (Iwakiri et al. 2023). DoGs are commonly detected based on read abundance downstream from annotated transcription end sites (TESs) relative to upstream gene body expression (Wiesel et al. 2018; Roth et al. 2020; Iwakiri et al. 2023). Because DoGs are typically less abundant than the corresponding processed transcripts, they may be under-detected in data sets prepared using hiAGPC, which still contain abundant readily extractable RNAs.

**FIGURE 3.**
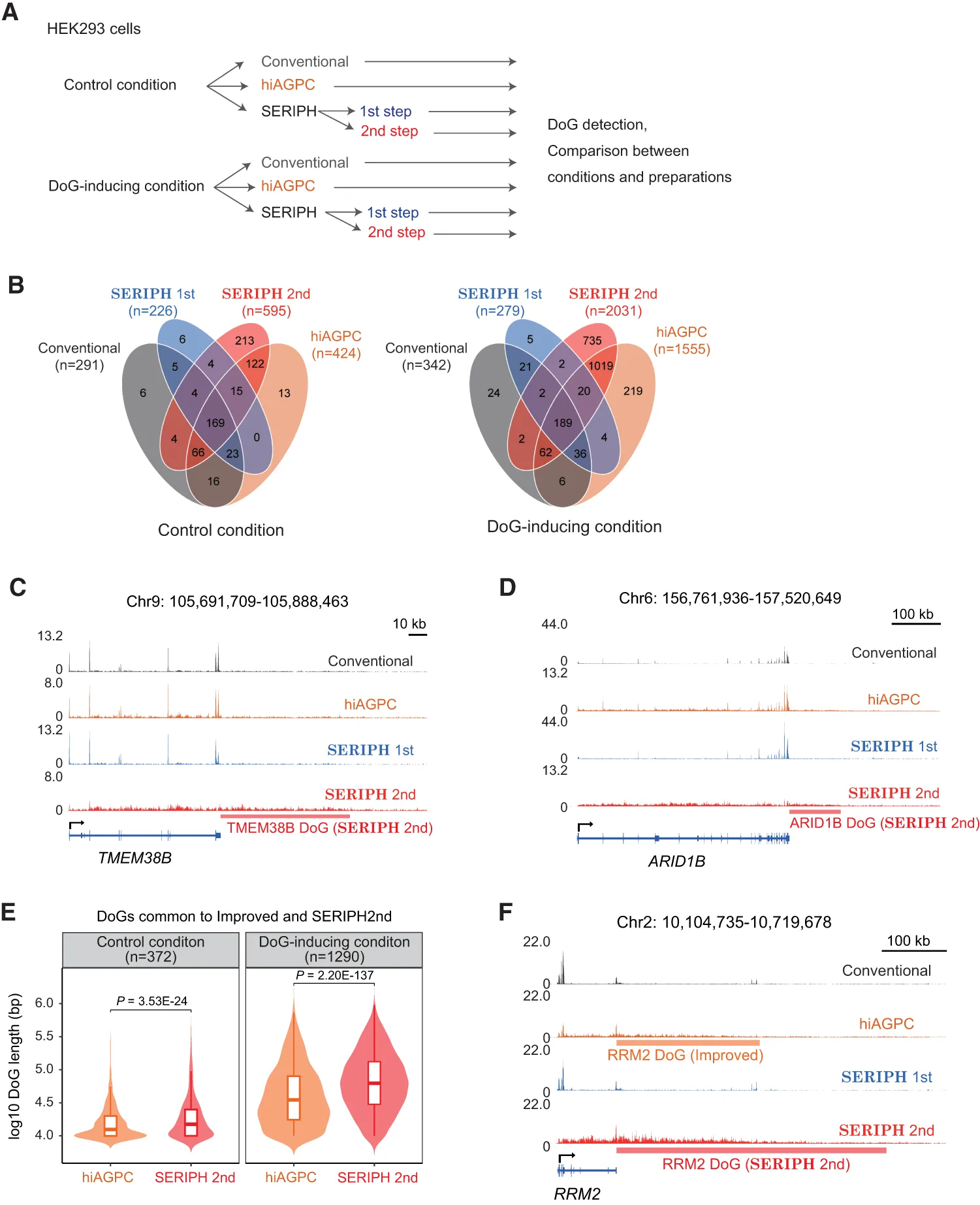
SERIPH enhances detection and genomic extent of transcriptional readthrough (DoG) transcripts. (*A*) Experimental design for comparative DoG detection. Total RNA was extracted under control and modified DoG-inducing conditions using conventional, hiAGPC, and SERIPH methods, followed by short-read RNA sequencing. DoGs were defined based on read coverage downstream from annotated transcription end sites (TESs) relative to upstream gene body expression. (*B*) Number of genes producing detectable DoG transcripts under control and DoG-inducing conditions across extraction methods. Only reproducible DoGs detected in both biological replicates are shown. Venn diagrams indicate shared and method-specific DoG-producing genes. (*C*) Genome browser view of the *TMEM38B* locus, an example of a DoG detected only in the SERIPH 2nd-step fraction under the control condition. Normalized coverage tracks are shown with replicates overlaid. Coverage tracks were group-autoscaled within conventional/SERIPH 1st-step, and separately within hiAGPC/SERIPH 2nd-step preparations. The bar beneath the SERIPH 2nd-step track indicates the DoG region defined from the SERIPH 2nd-step RNA-seq data set. (*D*) Genome browser view of the *ARID1B* locus, an example of a DoG detected only in the SERIPH 2nd-step fraction under the DoG-inducing condition. Coverage tracks are displayed as in *C*. (*E*) Comparison of DoG region lengths detected by the hiAGPC method and SERIPH 2nd-step fraction. Only reproducible DoGs detected in both replicates and common to both the hiAGPC and SERIPH 2nd-step data sets were included. For each DoG, lengths were averaged across replicates prior to log_10_ transformation. Violin plots show the distribution of log_10_-transformed DoG lengths; box plots indicate interquartile range and median. The number of DoGs analyzed in each condition is indicated in the facet label. Statistical significance was assessed using a paired Wilcoxon signed-rank test within each condition. (*F*) Genome browser view of the *RRM2* locus, an example of a DoG region that is longer in the SERIPH 2nd-step fraction than in hiAGPC. Coverage tracks are displayed as in *C*, and bars indicate the corresponding DoG regions.

We therefore compared DoG detection across conventional AGPC extraction, hiAGPC, and SERIPH preparations under control and modified DoG-inducing conditions (200 mM sorbitol with 100 μg/mL cycloheximide for 9 h; Fig. 3A; Supplemental Fig. S4; Supplemental Table S2). This condition enhanced and sustained DoG production, enabling robust detection across extended time windows. However, because full characterization of this condition is beyond the scope of the present study, we focus here on its utility for comparative evaluation of extraction methods.

Consistent with previous findings, hiAGPC increased the number of detectable DoGs relative to the conventional method under both control and the DoG-inducing conditions (Fig. 3B). The SERIPH 2nd-step fraction further increased DoG detection by ∼1.3-fold compared with hiAGPC, from 424 to 595 under control conditions and from 1555 to 2031 under DoG-inducing conditions (Fig. 3B). For example, *TMEM38B* and *ARID1B* DoGs were detected only in the SERIPH 2nd-step fraction under control and DoG-inducing conditions, respectively (Fig. 3C,D).

Moreover, among DoG-producing genes detected by both hiAGPC and SERIPH, downstream readthrough regions identified by SERIPH were significantly longer than those detected by hiAGPC (Fig. 3E), as exemplified by *RRM2* DoG (Fig. 3F). This likely reflects more efficient enrichment by SERIPH of low-abundance, semi-extractable DoG transcripts, allowing faint readthrough signals to be captured over greater genomic distances.

Notably, we frequently observed concomitant enrichment of unspliced pre-mRNAs and DoG transcripts in the SERIPH 2nd-step fraction, accompanied by a relative depletion of spliced exon peaks (Fig. 3C,D,F). These features are further analyzed below (see Fig. 5).

### Selective separation of semi-extractable DoGs from extractable mRNAs by SERIPH

We next examined whether SERIPH can separate transcripts derived from the same genomic locus based on their extractability. DoG-producing loci provide a useful model because they generate both semi-extractable readthrough transcripts and readily extractable processed mRNAs.

**FIGURE 4.**
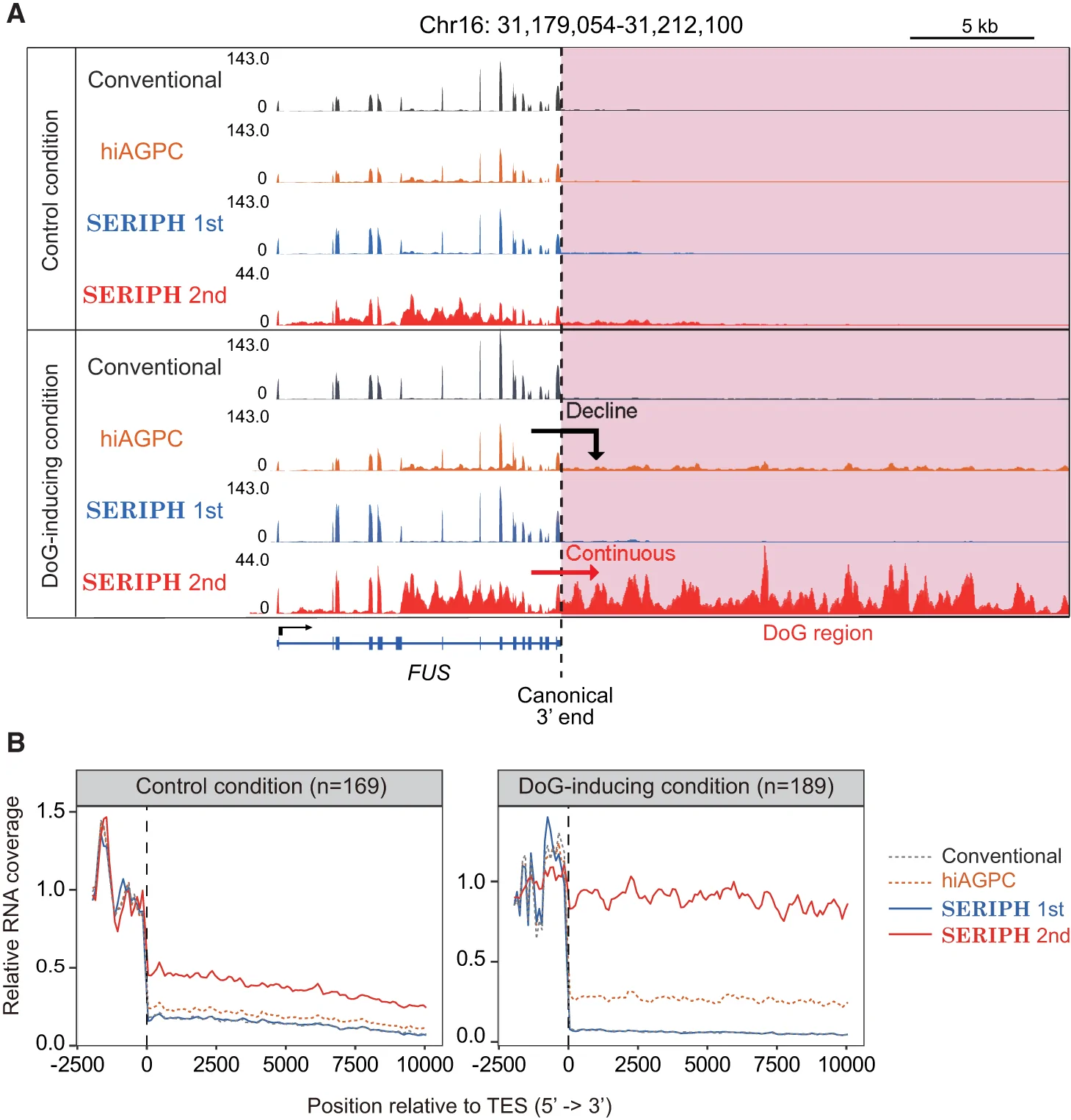
Selective separation of semi-extractable DoG transcripts from extractable mRNAs at shared genomic loci. (*A*) Genome browser view of the DoG-producing *FUS* locus under the modified DoG-inducing condition. Normalized coverage tracks are shown for conventional, hiAGPC, SERIPH 1st-step, and SERIPH 2nd-step RNA preparations, with biological replicates overlaid. Coverage tracks were autoscaled independently for each sample. The annotated TES is indicated by a black dashed line. In hiAGPC, coverage drops sharply downstream from the TES, whereas the SERIPH 2nd-step fraction maintains continuous coverage into the downstream DoG region. (*B*) Metagene analysis centered on annotated TESs of DoG-producing genes. Profiles were generated for DoGs common to all RNA preparations within each condition (*n* = 169 and 189 under control and DoG-inducing conditions, respectively). Strand-aware windows from −2 to +10 kb relative to the TES were divided into 100 bp bins, normalized to the mean signal in the 2 kb upstream region of TES, and averaged across genes. Biological replicates were averaged for visualization. The dashed vertical line indicates the TES (position 0).

We first examined the *FUS* locus, a highly expressed and well-characterized DoG-producing gene (Arnold et al. 2021; Iwakiri et al. 2023), under the modified DoG-inducing condition (Fig. 4A). In libraries prepared using hiAGPC, read coverage dropped sharply immediately downstream from the annotated 3′ end, and the much less abundant downstream readthrough transcripts contributed minimal signal, consistent with dominance by highly abundant, extractable mRNAs (Fig. 4A). In contrast, RNA from the SERIPH 2nd-step fraction showed continuous read coverage extending beyond the canonical gene end into the downstream region, indicating efficient enrichment of DoG transcripts (Fig. 4A). RT–qPCR analysis normalized to spliced *FUS* mRNA further supported enrichment of DoG transcripts in the SERIPH 2nd-step fraction compared with both the 1st-step and hiAGPC preparations (Supplemental Fig. S5A,B).

Similar patterns were observed at other DoG-producing loci, including *TMEM38B*, *ARID1B*, and *RRM2* (Fig. 3C,D,F). To generalize these observations, we performed a metagene analysis centered on TESs (Fig. 4B). DoG-producing genes detected in all preparations were analyzed (169 under control conditions and 189 under DoG-inducing conditions). Although hiAGPC showed moderately higher downstream coverage than conventional and SERIPH 1st-step preparations, coverage declined steeply across the TES. In contrast, the SERIPH 2nd-step fraction exhibited a smaller reduction across the TES and maintained more continuous read density into downstream regions. This feature was more pronounced under DoG-inducing conditions but remained detectable under control conditions.

Together, these findings demonstrate that SERIPH resolves transcript populations with distinct extractability properties arising from the same genomic loci, preferentially enriching semi-extractable DoG transcripts while depleting abundant processed mRNAs.

### SERIPH exposes extractability-linked transcript heterogeneity associated with RNA processing states

In the above analyses, SERIPH enrichment of DoG transcripts was frequently accompanied by accumulation of intronic reads at *TMEM38B*, *ARID1B*, *RRM2*, and *FUS* loci (Figs. 3C,D,F and 4A), indicative of intron-retaining or partially processed transcripts. At the *FUS* locus, increased DoG detection coincided with elevated retention of multiple downstream introns (Fig. 4A). Consistently, RT–qPCR normalization to unspliced *FUS* transcripts spanning Exon 7 and Intron 7 diminished the apparent enrichment of DoG-region signals in hiAGPC and SERIPH 2nd-step preparations (Supplemental Fig. S5A,C), suggesting that intron retention and readthrough occur within the same extractability-defined population at this locus.

**FIGURE 5.**
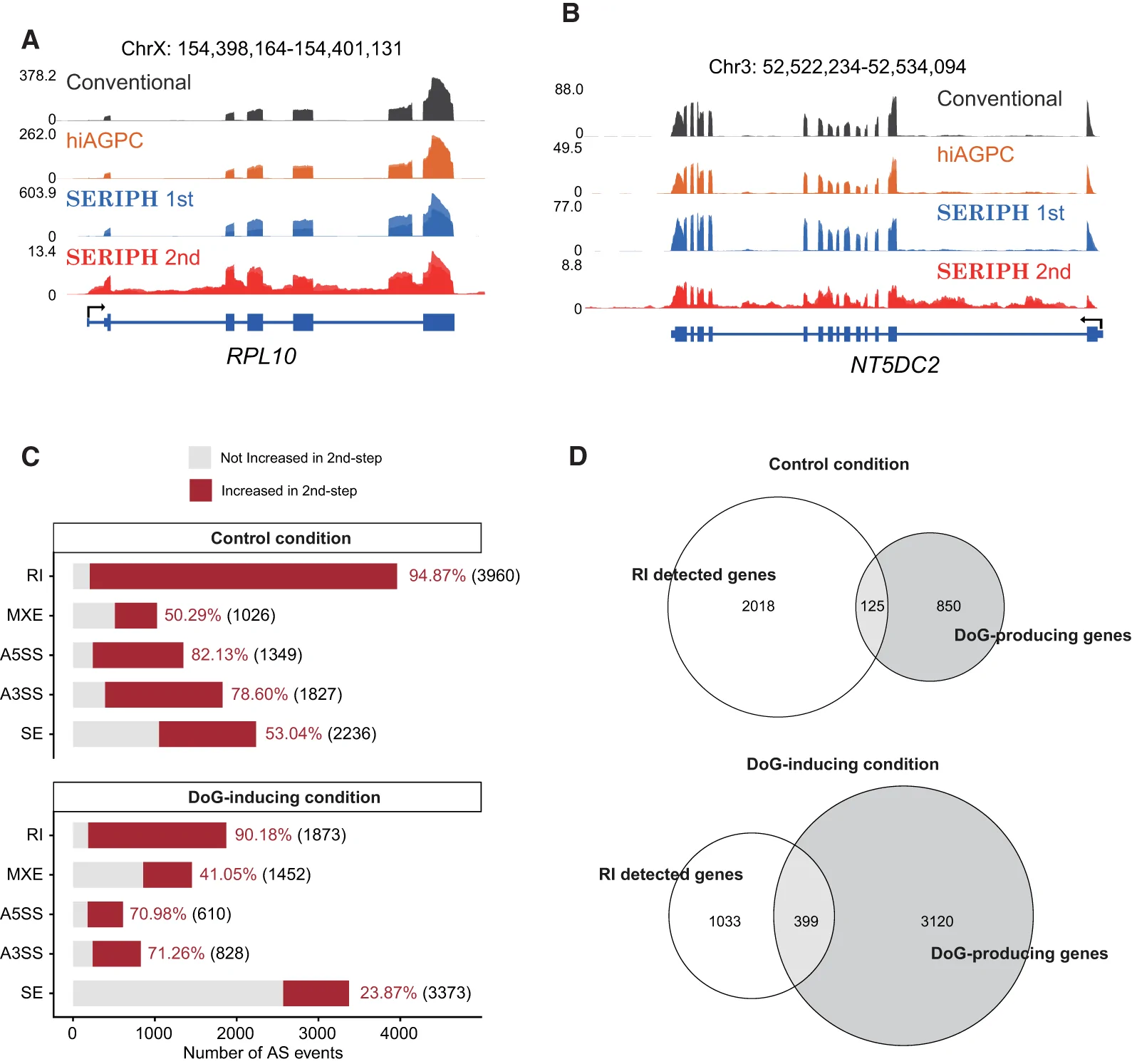
SERIPH enrichment reveals extractability-associated intron retention beyond canonical DoG loci. (*A*, *B*) Genome browser views of the *RPL10* locus (*A*) and the *NT5DC2* locus (*B*). Normalized coverage tracks are shown with biological replicates overlaid. Coverage tracks were autoscaled independently for each sample. Arrows indicate transcription direction. (*C*) Genome-wide analysis of alternative splicing events comparing the SERIPH 2nd-step and 1st-step fractions using rMATS. Alternative splicing events were required to have ≥10 total junction reads in both fractions and ≥5 inclusion junction reads in the 2nd-step fraction and were filtered at adjusted *P* < 0.05. Events are summarized by splicing type (RI, retained intron; MXE, mutually exclusive exons; A5SS, alternative 5′ splice site; A3SS, alternative 3′ splice site; SE, skipped exon). The total number of detected events for each splicing category is indicated in parentheses. Among these, events significantly increased in the 2nd-step fraction (adjusted *P* < 0.05 and IncLevelDifference < −0.1) are highlighted in red, with the corresponding percentages indicated. (*D*) Overlap between genes with retained intron (RI) events enriched in the SERIPH 2nd-step fraction and DoG-producing genes. Venn diagrams are shown for the control (*top*) and DoG-inducing (*bottom*) conditions. RI-enriched genes were defined as genes harboring RI events significantly increased in the 2nd-step fraction (adjusted *P* < 0.05; IncLevelDifference < −0.1), as determined in *C*. DoG-producing genes were defined based on reproducible detection of downstream-of-gene (DoG) transcripts in SERIPH 2nd-step fraction within each condition (see Fig. 3). Numbers indicate gene counts in each region.

Importantly, intronic read enrichment was not restricted to canonical DoG-producing loci. At the *RPL10* and *NT5DC2* loci, neither of which showed evident transcriptional readthrough, intronic enrichment was observed, with nearly all introns enriched at *RPL10* and a subset retained and enriched at *NT5DC2* (Fig. 5A,B). A related feature was also observed at the *FUS* locus: under the control condition, coverage in the SERIPH 2nd-step fraction remained higher over the gene body and retained introns than over the downstream DoG region (Fig. 4A), suggesting that semi-extractability at this locus may also arise from mechanisms distinct from downstream readthrough transcription. Notably, *FUS* transcripts retaining introns 6 and 7 were recently reported to form nuclear RNA condensates enriched for splicing factors and YTHDC1 and to facilitate posttranscriptional *FUS* splicing in an m6A-dependent manner (Huang et al. 2025). These introns were enriched in our SERIPH 2nd-step data set, suggesting that SERIPH can capture RNA populations associated with defined condensate-linked processing states.

To obtain a broader gene-level view of transcript processing states, we classified genes into five categories based on exon and intron read patterns: mature mRNA-like, partially spliced, pre-mRNA-like, unclassified, and low-coverage (Supplemental Fig. S6A; Supplemental Table S3). The unclassified category included genes that did not clearly fit the predefined categories, including single-intron genes for which partial versus global intron retention could not be distinguished. This analysis showed that partially spliced, pre-mRNA-like, and unclassified genes were increased in hiAGPC and were more prominently represented in the SERIPH 2nd-step fraction, whereas mature mRNA-like genes were reduced in hiAGPC and were even more markedly reduced in the SERIPH 2nd-step fraction.

We further examined the relationship between these processing categories and RNA extractability-related classifications, namely semi-extractability and SERIPH 2nd-step enrichment (Supplemental Fig. S6B). In the conventional and SERIPH 1st-step fractions, mature mRNA-like genes accounted for the majority of genes regardless of extractability-related classification. In contrast, in hiAGPC and the SERIPH 2nd-step fraction, the proportion of mature mRNA-like genes was markedly reduced, particularly among genes classified as SERIPH 2nd-step enriched, and categories representing less processed states were more apparent.

We next asked whether these extractability-associated processing states were related to gene architecture. Genes in less processed categories that were increased in the SERIPH 2nd-step fraction tended to be longer, contain larger intronic regions, and have more introns (Supplemental Fig. S7A; Supplemental Table S3). Similar tendencies were observed for genes classified as semi-extractable or enriched in the SERIPH 2nd-step fraction (Supplemental Fig. S7B,C). Although short-read RNA-seq cannot fully resolve complete transcript isoforms, these gene-level analyses support the idea that SERIPH enrichment is associated with incompletely processed transcript populations.

To further evaluate this association at the splicing-event level using junction reads, we performed genome-wide rMATS analysis comparing the SERIPH 2nd-step and 1st-step fractions. This analysis identified numerous alternative splicing events (Supplemental Table S4). Retained intron events accounted for the largest class of events increased in the 2nd-step fraction under both control and DoG-inducing conditions, and ∼90% of detected RI events showed increased inclusion in this fraction (Fig. 5C). RI-enriched genes partially overlapped with DoG-producing genes, but many were independent of DoG detection (Fig. 5D). These findings indicate that SERIPH-associated intronic enrichment reflects not only co-enrichment of DoG transcripts and retained introns, but also preferential recovery of intron-retaining transcripts from loci independent of transcriptional readthrough, consistent with our previous observation that unspliced transcript-focused analysis expands the set of 2nd-step-enriched targets (Supplemental Fig. S3D–F).

Collectively, these results suggest that RNA extractability reflects broader RNA processing states, including intron retention and partial or incomplete splicing, rather than transcriptional readthrough alone. By resolving extractability-linked transcript heterogeneity at the transcript level, SERIPH reveals RNA populations that remain obscured in data sets dominated by highly abundant mRNA species.

## DISCUSSION

In this study, we developed SERIPH, a simple interphase-based RNA fractionation protocol that separates readily extractable RNAs from semi-extractable RNAs (seRNAs), thereby improving their representation in transcriptome-wide analyses. Unlike the Chujo 2017 method, which improves recovery but yields a mixed RNA pool (Chujo et al. 2017), SERIPH was designed as a fractionation strategy to enrich semi-extractable RNAs that are typically underrepresented in conventional transcriptomic workflows.

A major advantage of SERIPH is that it resolves RNA populations according to extractability, even when distinct transcript species arise from the same genomic locus. This is illustrated by DoG-producing genes, in which SERIPH enriches semi-extractable readthrough transcripts while depleting the corresponding processed mRNAs. Our data further suggest that a single locus can give rise to transcripts with distinct extractability properties and splicing states. Consistent with this view, gene-level classification of exon and intron read patterns indicated that SERIPH 2nd-step enrichment is associated with incompletely processed transcript populations, including transcripts with intron-retaining or partially spliced features.

By reducing the background of abundant extractable RNAs, SERIPH also enhances detection of weakly semi-extractable transcripts that fall below conventional threshold-based classifications. This suggests that the apparent seRNA repertoire is strongly influenced by extraction and detection sensitivity. Because SERIPH requires no specialized equipment or reagents beyond standard AGPC chemistry, it should be readily applicable to diverse sample types, including tissue specimens, and should therefore help expand the detectable seRNA repertoire.

These advances may help address important open questions, such as how semi-extractability relates to functional boundaries among RNA classes, whether some newly detected seRNAs can function as bona fide architectural RNAs, and what molecular mechanisms underlie reduced extractability. Importantly, semi-extractability should not be interpreted as defining a single functional RNA class. Although this study highlights arcRNAs, DoG transcripts, and incompletely processed RNAs as representative SERIPH-enriched examples, seRNAs are likely to comprise a broad and heterogeneous population, and not all arcRNAs or incompletely processed RNAs necessarily exhibit semi-extractability. Moreover, additional RNA species not fully captured by the present analysis framework, including repetitive-element-derived RNAs, may also contribute to the SERIPH-enriched fraction and should be examined in future studies. The expanded repertoire of semi-extractable RNAs and the transcript-level information provided by SERIPH should be valuable for integrative analyses with complementary approaches, such as long-read sequencing and identification of associated factors. Such analyses are expected to deepen our understanding of the RNA features and molecular interaction states underlying semi-extractability, and to clarify its biological significance in individual transcript contexts.

At the same time, SERIPH has limitations. As a fractionation-based method, it is better suited to enrichment- and property-focused analyses than to estimation of absolute cellular RNA abundance. In this respect, the hiAGPC method remains more appropriate for quantitative expression profiling, whereas SERIPH provides complementary value by enriching RNA populations that are diluted or obscured in conventional RNA preparations. In addition, enrichment in the SERIPH 2nd-step fraction reflects both transcript abundance and extractability. As illustrated by MALAT1, highly abundant RNAs may still be detected in the 2nd-step fraction even without strong semi-extractable behavior.

We also did not directly test whether SERIPH or the hiAGPC method can be applied to other extraction systems, such as column-based purification methods or reagents like NucleoZOL that do not generate a classical interphase. In principle, semi-extractability may still be assessable in such systems if the underlying interactions are sufficiently preserved during homogenization and extraction and if semi-extractable RNAs can be recovered through an appropriate physical disruption. Whether this is feasible in practice remains to be determined.

Taken together, SERIPH provides a simple but conceptually distinct framework for property-based RNA fractionation. By enabling sensitive and selective access to RNA populations that are underrepresented in conventional RNA preparations, SERIPH should expand the detectable seRNA repertoire, facilitate molecular characterization of heterogeneous seRNA populations, and accelerate systematic investigation of the relationships among RNA extractability, RNA processing, nuclear organization, and transcriptional regulation in stress responses and disease.

## MATERIALS AND METHODS

### Cell lines and culture conditions

HEK293 and HeLa cells were maintained in Dulbecco's Modified Eagle Medium (DMEM) supplemented with 10% heat-inactivated fetal bovine serum (FBS; Sigma) at 37°C in a humidified incubator with 5% CO_2_. For downstream-of-gene (DoG) transcripts induction, HEK293 cells were seeded at 1.0 × 10^6^ cells per 10 cm dish and cultured for 48 h. The medium was then replaced with DMEM + 10% FBS containing 200 mM sorbitol (FUJIFILM Wako Pure Chemical Corporation) and 100 µg/mL cycloheximide (Nacalai Tesque), and cells were incubated for an additional 9 h. For control samples, the medium was replaced with DMEM + 10% FBS lacking sorbitol and CHX.

### RNA fractionation based on semi-extractability (SERIPH)

seRNAs were enriched using SERIPH (semi-extractable RNA isolation from the interphase). A detailed step-by-step SERIPH workflow is provided in Supplemental Figure S1. Briefly, cells were lysed in TRI reagent (1 × 10^6^ cells per 1 mL), followed by chloroform addition (200 µL per 1 mL lysate). After phase separation by centrifugation (15,000*g*, 15 min, 4°C), the upper aqueous phase containing extractable RNAs was collected as the “SERIPH 1st-step fraction.” The interphase was recovered, washed three times with freshly phase-separated TRI Reagent/chloroform mixture prepared from 1 mL TRI reagent and 200 µL chloroform. The washed interphase was resuspended in 1 mL TRI reagent, and homogenized by vortexing, with brief 20-gauge needle shearing if required. The sample was then incubated at 55°C for 10 min, followed by addition of chloroform (200 µL), vortexing, and centrifugation (20,380*g*, 15 min, 4°C). The resulting aqueous phase was collected as the seRNA-enriched “SERIPH 2nd-step fraction.” For head-to-head comparisons, conventional AGPC and hiAGPC extractions were performed in parallel on the same samples.

### RNA purification, DNase treatment, reverse transcription and RT–qPCR

RNAs were precipitated with isopropanol in the presence of glycogen, washed with 75% ethanol, and resuspended in RNase-free water. For downstream normalization of low-abundance SERIPH 2nd-step samples, in vitro transcribed *Cypridina* luciferase mRNA spike-ins were added. A fragment corresponding to nucleotides 1–1000 of the *Cypridina* luciferase coding sequence was PCR-amplified from pMCS–*Cypridina* Luc (Thermo Fisher Scientific). A T7 promoter sequence and a poly(A)_59_ stretch were appended to the fragment, which was then cloned into the Zero Blunt TOPO vector (Thermo Fisher Scientific). Using this plasmid as a template, the T7 promoter–containing *Cypridina* luciferase fragment was PCR-amplified and subjected to in vitro transcription with T7 RNA polymerase (Promega) to generate spike-in RNA.

Total RNA was treated with RQ1 RNase-Free DNase (Promega) and re-extracted with acid phenol–chloroform–isoamyl alcohol (25:24:1 Mixed, pH 5.2; Nacalai Tesque). The recovered aqueous phase was ethanol-precipitated, washed with 75% ethanol and dissolved in RNase-free water. An aliquot containing exactly 400 ng of RNA was adjusted to 4 µL, supplemented with 2.5 ng of *Cypridina* luciferase spike-in RNA, and reverse transcribed with random hexamers using the High-Capacity cDNA Reverse Transcription Kit (Applied Biosystems) according to the manufacturer's instructions. qPCR was performed with KAPA SYBR Fast qPCR Kit (KAPA Biosystems) on a Roche Light Cycler 488. Relative RNA levels were quantified using standard curve–based relative quantification and normalized to the *Cypridina* luciferase spike-in control. Primer sequences are listed in Supplemental Table S5.

### Short-read RNA-seq library construction and sequencing

Short-read RNA-seq libraries were prepared and sequenced by Macrogen. Briefly, to retain nonpolyadenylated species, libraries were prepared from rRNA-depleted total RNA (100–500 ng for SERIPH 1st; 5–50 ng for SERIPH 2nd) using Illumina Stranded Total RNA Prep with Ribo-Zero Plus (Illumina). Libraries were quality-controlled (Agilent Technologies 2100 Bioanalyzer; Agilent Technologies) and quantified by Qubit Fluorometer (Thermo Fisher Scientific). Sequencing was performed on an Illumina (NovaSeqX; Illumina) to generate paired-end 150 bp reads. Nominal depths were 20–60 million read pairs per sample.

### Read processing and quantification

Sequencing reads were preprocessed with fastp (v0.25.0) using the options ‐‐detect_adapter_for_pe, ‐‐qualified_quality_phred 20, and ‐‐length_required 30 to remove adapter sequences and discard low-quality and short reads (Chen et al. 2018). Filtered reads were first aligned to human 45S rDNA (KY962518.1) using Bowtie2 (v2.5.4) (Langmead and Salzberg 2012). Reads unmapped to rDNA were subsequently aligned to the human genome (GRCh38 primary assembly) with GENCODE v49 gene annotation using STAR (v2.7.11b) (Dobin et al. 2013; Mudge et al. 2025). For STAR alignment, ‐‐outFilterMultimapNmax 1 was specified so that only uniquely mapped reads were retained. The results from all processing steps were summarized and visualized using MultiQC (v1.29) for integrated quality assessment (Ewels et al. 2016).

### Detection of readthrough (DoG) transcripts

DoGs were identified from the alignments using ARTDeco (Roth et al. 2020) with the following parameters: -read-in-threshold -1, -read-in-fpkm 1, -min-dog-len 10,000, -dog-window 5000, and -min-dog-coverage 0.5. These parameters were empirically determined on the basis of the DoG detection profile at *RPS12* to facilitate detection of DoG extension across genomic regions containing repetitive elements, such as L1Hs, where stretches of uniquely mapped reads are often interrupted. To limit the analysis to relatively well-characterized genes, only GENCODE v49 entries containing either “protein_coding” or “lncRNA” were included. Entries with automatically assigned gene_name values beginning with “ENSG” or “LINC” were excluded; this filtering was applied to gene_name, not gene_id. This filtering was introduced to reduce analytical ambiguity, because transcriptional readthrough from upstream genes often complicates expression estimates and interpretation of downstream entries in DoG-producing conditions. To detect readthrough transcription downstream from canonical transcription end sites, entries annotated with the “readthrough” tag were also excluded. For analysis of unspliced RNA, ARTDeco was applied to nonexonic regions of the filtered GENCODE v49 gene set. Statistical comparisons between the two conditions were performed through ARTDeco using DESeq2 v1.20 (Love et al. 2014).

### Identification of semi-extractable and SERIPH-enriched RNAs

“Semi-extractability” was defined from conventional AGPC versus hiAGPC data sets by modeling log_2_FC (hiAGPC/conventional AGPC) with DESeq2 (Benjamini–Hochberg FDR < 0.05; typical |log_2_FC| ≥ 1). “2nd-step enrichment” was assessed by differential analysis of SERIPH 2nd versus SERIPH 1st (DESeq2; FDR < 0.05; typical log_2_FC ≥ 1). The overlap between 2nd-step-enriched RNAs and the semi-extractable callset was used to quantify recovery of known semi-extractables and to identify weak candidates newly captured by SERIPH.

### Coverage-based classification of RNA processing states

To classify RNA processing states transcriptome-wide, we performed a gene-level exon/intron coverage analysis using strand-specific paired-end total RNA-seq data. Exons from all annotated isoforms of each gene were merged to define union exon regions, and introns were defined as the intervening regions between adjacent merged exons; introns shorter than 100 nt were excluded. Intron number and total intronic length used in downstream gene-feature analyses were calculated after this filtering step. Coverage was computed using a custom pysam-based (https://github.com/pysam-developers/pysam) approach that inferred transcript strand from read orientation and library type and counted only aligned read blocks, thereby excluding CIGAR N skipped regions from intron coverage.

For each gene, we calculated length-weighted mean exon and intron coverage, gene intronic fraction (GIF), exon–pre-mRNA preference score (EPPS), intron uniformity score (IUS), and splice-junction support score (SJSS). GIF was defined as intron coverage divided by the sum of exon and intron coverage. EPPS was defined as the log_2_ ratio of exon to intron coverage. IUS was defined as one minus the coefficient of variation of intron coverage across introns within the same gene. SJSS was defined as the fraction of gene-overlapping read pairs containing a spliced CIGAR operation.

Genes were classified using operational thresholds. Genes with exon plus intron coverage < 1.0 were labeled as *low_coverage*. Genes with GIF < 0.15, EPPS > 1.5, and exon coverage ≥1.0 were classified as *mature_mRNA_like*. Genes with GIF ≥ 0.35, EPPS < 0.75, IUS ≥ 0.30, and SJSS < 0.30 were classified as pre_mRNA_like. Genes with 0.15 ≤ GIF < 0.70 and SJSS ≥ 0.10 were classified as *partially_spliced*. Genes passing the coverage threshold but not satisfying any of these criteria were labeled as *unclassified*. Because IUS requires at least two analyzable introns, single-intron genes were conservatively retained as *unclassified* when they did not meet the other category definitions, even if intronic signal was visually apparent in genome-browser tracks.

### Alternative splicing analysis (rMATS)

Alternative splicing analysis was performed using rMATS v4.1.2 (Shen et al. 2014). Input BAM files were generated as described in the above section. Paired-end reads (150 bp) were analyzed using the GENCODE v49 annotation. rMATS was run with ‐‐variable-read-length, ‐‐allow-clipping, and ‐‐libType fr-firststrand, specifying a stranded paired-end library configuration. Splicing events were quantified using junction counts only. Events were filtered to require ≥10 total junction reads in both fractions and ≥5 inclusion junction reads in the 2nd-step fraction. Differential alternative splicing events were defined based on an adjusted *P* < 0.05. Events significantly increased in the SERIPH 2nd-step fraction were further defined by an IncLevelDifference <−0.1. The following splicing event types were analyzed: retained intron (RI), skipped exon (SE), mutually exclusive exons (MXE), alternative 5′ splice site (A5SS), and alternative 3′ splice site (A3SS).

### Data visualization

Genome browser tracks were visualized using SparK v2.6.2 (Kurtenbach and William Harbour 2019). bigWig files were converted to bedGraph format using bigWigToBedGraph (UCSC Kent utilities). Statistical analyses and graphical visualizations were performed in R using ggplot2 (Ginestet 2011).

### DATA DEPOSITION

Raw and processed RNA-seq data are deposited in GEO under accession GSE319926.

## SUPPLEMENTAL MATERIAL

Supplemental material is available for this article.

## Figures and Tables

**Naoko Fujiwara:**
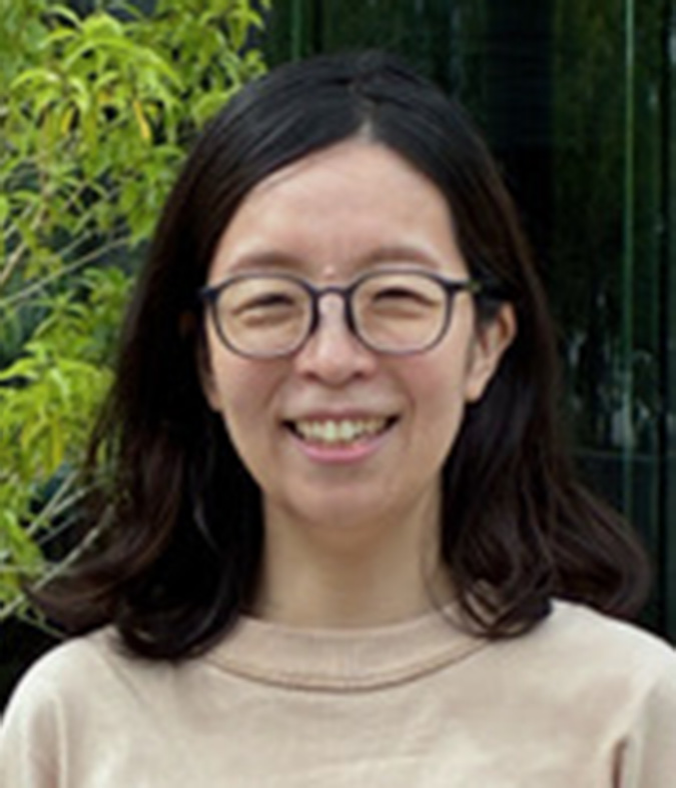


## MEET THE FIRST AUTHOR

***Meet the First Author(s)* is an editorial feature within *RNA*, in which the first author(s) of research-based papers in each issue have the opportunity to introduce themselves and their work to readers of *RNA* and the RNA research community. Naoko Fujiwara is the first author of this paper, “SERIPH: a two-step extraction protocol for selective enrichment of semi-extractable RNAs.” Naoko carried out this research as an assistant professor in the Hirose laboratory at The University of Osaka. Her research focuses on the functional analysis of long noncoding RNAs (lncRNAs), with particular interest in semi-extractable RNAs, architectural RNAs, and RNA-mediated nuclear organization.**

**What are the major results described in your paper and how do they impact this branch of the field?**

Transcriptome analyses depend on RNAs recovered by extraction, but not all cellular RNAs are extracted equally. Semi-extractable RNAs are poorly recovered by conventional AGPC extraction and are therefore underrepresented in standard RNA-seq data sets. This is important because RNA-seq does not directly measure all RNAs in a cell; it measures the RNAs that can be recovered by extraction. Semi-extractability is not only a technical limitation but may also reflect the molecular state of RNA in cells, such as strong interactions with proteins, chromatin, or nuclear structures.

Although previous improved extraction methods enhanced the recovery of semi-extractable RNAs, they also recovered abundant readily extractable RNAs, making focused analysis difficult. In this study, we developed SERIPH, a simple two-step RNA preparation method that separates cellular RNAs by extractability and efficiently enriches relatively minor semi-extractable RNA populations. By distinguishing RNA species with different extractability properties, even when they arise from the same genomic locus, SERIPH enables focused analysis of semi-extractable RNA populations that are otherwise difficult to resolve. Because SERIPH requires no specialized equipment and can be added to conventional AGPC-based workflows, it should be broadly applicable. We hope that SERIPH will help generate higher-resolution information on semi-extractable RNAs and promote future studies on the molecular mechanisms underlying RNA semi-extractability and the physiological functions of individual semi-extractable RNAs.

**What led you to study RNA or this aspect of RNA science?**

My interest in lncRNAs grew during my PhD work on the RNA exosome, when I noticed that a surprisingly diverse set of lncRNAs was degraded as substrates of this complex. These lncRNAs did not seem to be merely transcriptional noise; many appeared to be dynamically regulated. This experience made me appreciate the complexity and precision of lncRNA biology and lncRNA-mediated gene regulation. It also led me to ask how lncRNAs are controlled and how their molecular properties relate to their functions. This perspective eventually brought me to the study of architectural RNAs and RNA semi-extractability. Semi-extractability is certainly a technical issue, but at the same time, it may reflect strong molecular interactions or particular RNA states inside cells. Another aspect I find attractive about lncRNA research is that many methods are still developing. This makes the field challenging but also leaves room for creativity. I enjoy finding ways to overcome technical difficulties through methodological ideas and experimental adjustments, because these challenges often become starting points for new biological discoveries.

**During the course of these experiments, were there any surprising results or particular difficulties that altered your thinking and subsequent focus?**

A surprising result during this project was that SERIPH revealed a broader semi-extractable RNA population than I had initially expected. I initially thought that the main advantage of SERIPH would be to enrich known semi-extractable RNAs more cleanly by reducing contamination from abundant readily extractable RNAs. However, SERIPH also detected many weakly semi-extractable RNAs that had been overlooked by previous analyses, thereby expanding the detectable repertoire of semi-extractable RNAs. This finding made me realize that SERIPH can provide a more precise basis for exploring RNA features associated with semi-extractability. It also motivated my interest in applying SERIPH to more complex and heterogeneous samples, such as tissues, to further expand the semi-extractable RNA repertoire. I am also interested in combining SERIPH with complementary approaches, such as long-read sequencing, to obtain more detailed transcript-level information.

**What are some of the landmark moments that provoked your interest in science or your development as a scientist?**

One of the earliest landmarks in my interest in science was growing up in the countryside, surrounded by nature. As a child, I often observed plants, insects, and animals, fascinated by their diversity, forms, behaviors, and seasonal changes. I did not understand them in a scientific way at the time, but I was drawn to both the diversity of living things and the common principles that connect them.

Later, when I began to study natural science, I was deeply impressed that these childhood observations could be interpreted through biological principles. I found beauty in the process by which observation becomes understanding, and in realizing how diverse living systems can be connected by shared mechanisms. This perspective still influences my research today: I remain fascinated by carefully observing phenomena that may seem ordinary at first and asking what biological meaning and evolutionary history may lie behind them.
